# Supplementary material for: People with Primary Progressive Multiple Sclerosis Have a Lower Number of Central Memory T Cells and HLA-DR+ Tregs
Source: Cells. 2023 Jan 29;12(3):439. doi: 10.3390/cells12030439 (PMC9913799; doi:10.3390/cells12030439)
Supplement: Supplementary file 1 [file cells-12-00439-s001.zip › Suppl. Figures @20230127.pdf]

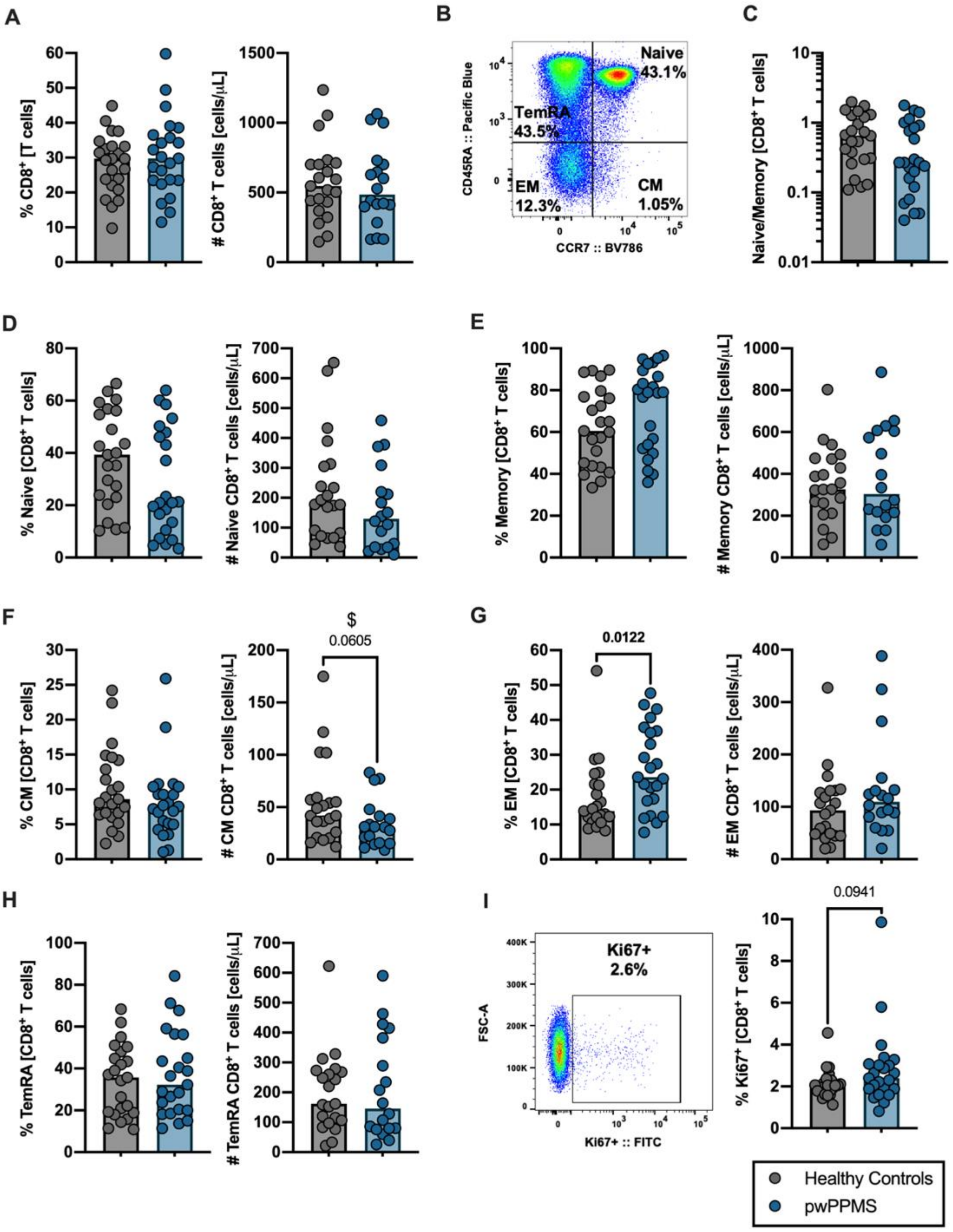

Supplementary Figure S1. pwPPMS display lower central memory CD8^{+} T cells number. Number and percentage of CD8^{+} T cells are represented for healthy controls (gray circles) and pwPPMS [blue circles; (**A**)]. Representative dot plot defining CD8^{+} T cell subpopulations according to the expression of CD45RA and CCR7 (**B**): naïve (CD45RA^{+}CCR7^{+}); central memory (CM; CD45RA^{-}CCR7^{+}), effector memory (EM; CD45RA^{-}CCR7^{-}); and terminally differentiated CD45RA-expressing memory T cells (TemRA; CD45RA^{+}CCR7^{-}). Ratio of naïve to memory CD8^{+} T cells where the total memory CD8^{+} T cells were considered the sum of CM, EM, and TemRA subsets (**C**). Percentage and number of CD8^{+} T cell subsets: naïve (**D**), total memory (**E**), CM (**F**), EM (**G**), and TemRA (**H**). Representative dot plot and percentage of proliferating (Ki67^{+}) CD8^{+} T cells (**I**). All graphs display a dot per individual, with the horizontal lines representing the groups' median. The parametric Student's *t*-test was performed in (**A**), (**C**), (**D**; percentage), (**E**) and (**H**; percentage); the non-parametric Mann-Whitney *U*-test in (**D**; number), (**F**), (**G**), (**H**; number) and (**I**). *P*-values were represented in the graph whenever <0.100, and differences were considered statistically significant for *p*-value <0.050. Statistical outputs and effect size calculations in **Supplementary Table S1**. $^{\$}$, stands for situations when significance is gained upon controlling for age and sex on the multiple linear regression models (**Supplementary Table S3)**.

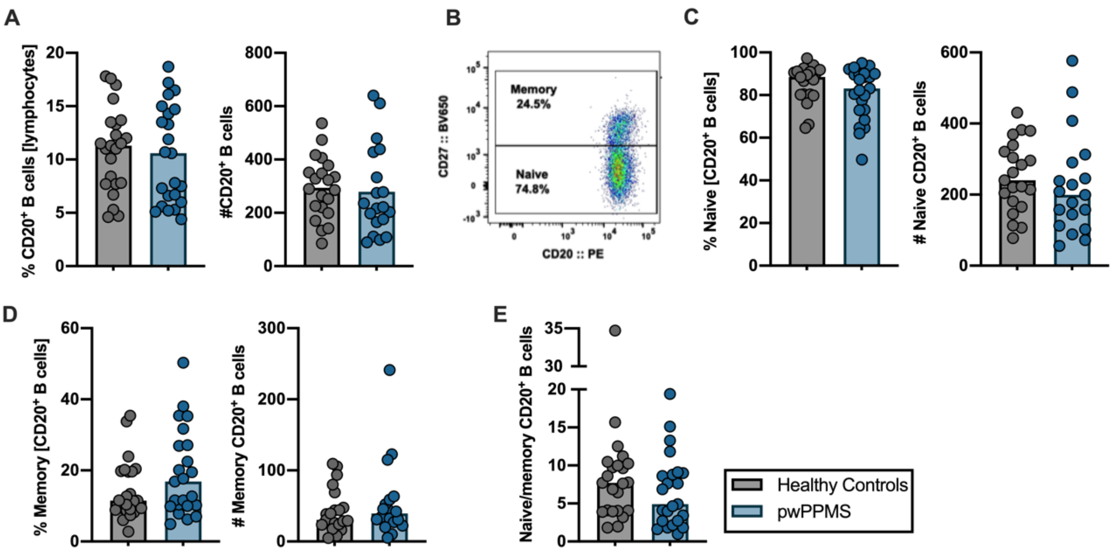

Supplementary Figure S2. pwPPMS present no alterations on B cell subsets**.** Number and percentage of B cells ($CD19^+$ $CD20^+$) are represented for healthy controls (gray circles) and pwPPMS [blue circles; (**A**)]. Representative dot plot defining B cell subpopulations according to the expression of CD27 (**B**): naïve ($CD19^+CD20^+CD27^-$) and memory ($CD19^+CD20^+CD27^+$). Percentage and number of B cell subsets: naïve (**C**), memory (**D**) and ratio of naïve to memory B cells (**E**). All graphs display a dot per individual, with the horizontal lines representing the groups' median. The parametric Student's *t*-test was performed in (**A,** number); the non-parametric Mann-Whitney *U*-test in (**A**; percentage), (**C**), (**D**) and (**E**). *P*-values were represented in the graph whenever <0.100, and differences were considered statistically significant for *p*-value <0.050. Statistical outputs and effect size calculations in **Supplementary Table S1** and **S4**.

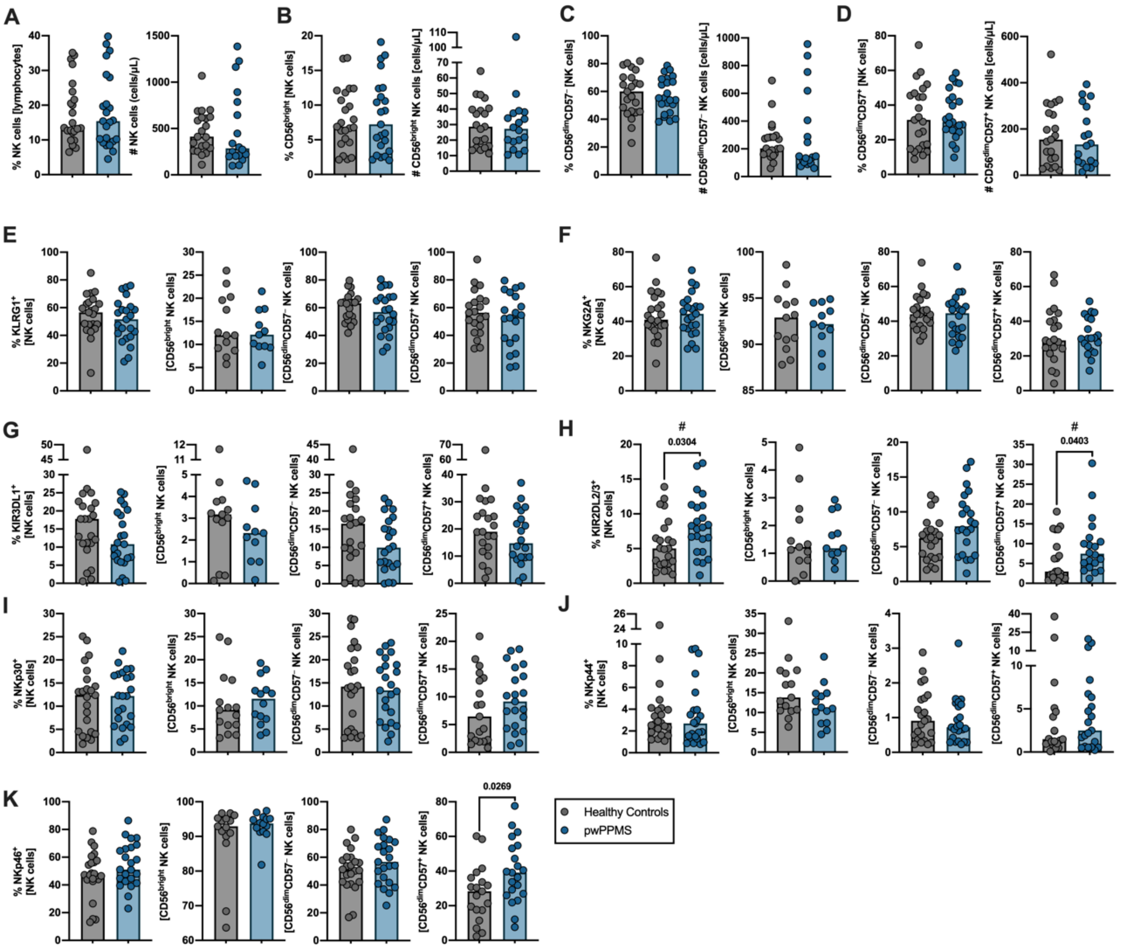

Supplementary Figure S3. pwPPMS display a higher percentage of the most mature NK cells expressing NKp46. Percentage and number of NK cells ($CD3^{-}CD56^{+}$) are represented for healthy controls (gray circles) and pwPPMS [blue circles; (**A**)]. Representation of percentage and number of NK cells subpopulations: $CD56^{bright}$ [most immature; (**B**)]; $CD56^{dim}CD57^{-}$ (**C**); and $CD56^{dim}CD57^{+}$ [most differentiated; (**D**)]. Percentage of cells expressing inhibitory receptors in total NK cells and on its three subsets: KLRG1 (**E**), NKG2A (**F**), KIR3DL1 (**G**) and KIR2DL2/3 (**H**). Percentage of cells expressing activating receptors in total NK cells and in its subsets: NKp30 (**I**), NKp44 (**J**), and NKp46 (**K**). All graphs display a dot per individual, with the horizontal lines representing the groups' median. The parametric Student's *t*-test was performed in (**A**), (**B-C**; percentage), (**D**), (**E**; NK cells, $CD56^{bright}$, $CD56^{dim}CD57^{-}$ [percentage] and $CD56^{dim}CD57^{+}$ [percentage]), (**F**; percentage), (**G**; $CD56^{dim}CD57^{-}$ [percentage] and $CD56^{dim}CD57^{+}$ [number]), (**H**; NK cells [percentage], $CD56^{bright}$ [percentage] and $CD56^{dim}CD57^{-}$ [percentage]), (**I**; percentage), (**J**; $CD56^{bright}$ [percentage]), (**K**; NK cells [percentage], $CD56^{dim}CD57^{-}$ [percentage] and $CD56^{dim}CD57^{+}$); the non-parametric Mann-Whitney *U*-test in (**B-C**; number), (**E**; $CD56^{dim}CD57^{-}$ [number] and $CD56^{dim}CD57^{+}$ [number]), (**F**; number), (**G**; NK cells, $CD56^{bright}$, $CD56^{dim}CD57^{-}$ [number] and $CD56^{dim}CD57^{+}$ [percentage]), (**H**; NK cells [number], $CD56^{bright}$ [number], $CD56^{dim}CD57^{-}$ [number] and $CD56^{dim}CD57^{+}$), (**I**; number), (**J**; NK cells, $CD56^{bright}$ [number], $CD56^{dim}CD57^{-}$ and $CD56^{dim}CD57^{+}$), (**K**; NK cells [number], $CD56^{bright}$ and $CD56^{dim}CD57^{-}$ [number]). *P*-values were represented in the graph whenever <0.100, and differences were considered statistically significant for *p*-value <0.050. Statistical outputs and effect size calculations in **Supplementary Table S1**. $^{\#}$, stands for situations when significance is lost upon controlling for age and sex on the multiple linear regression models (**Supplementary Table S6**).

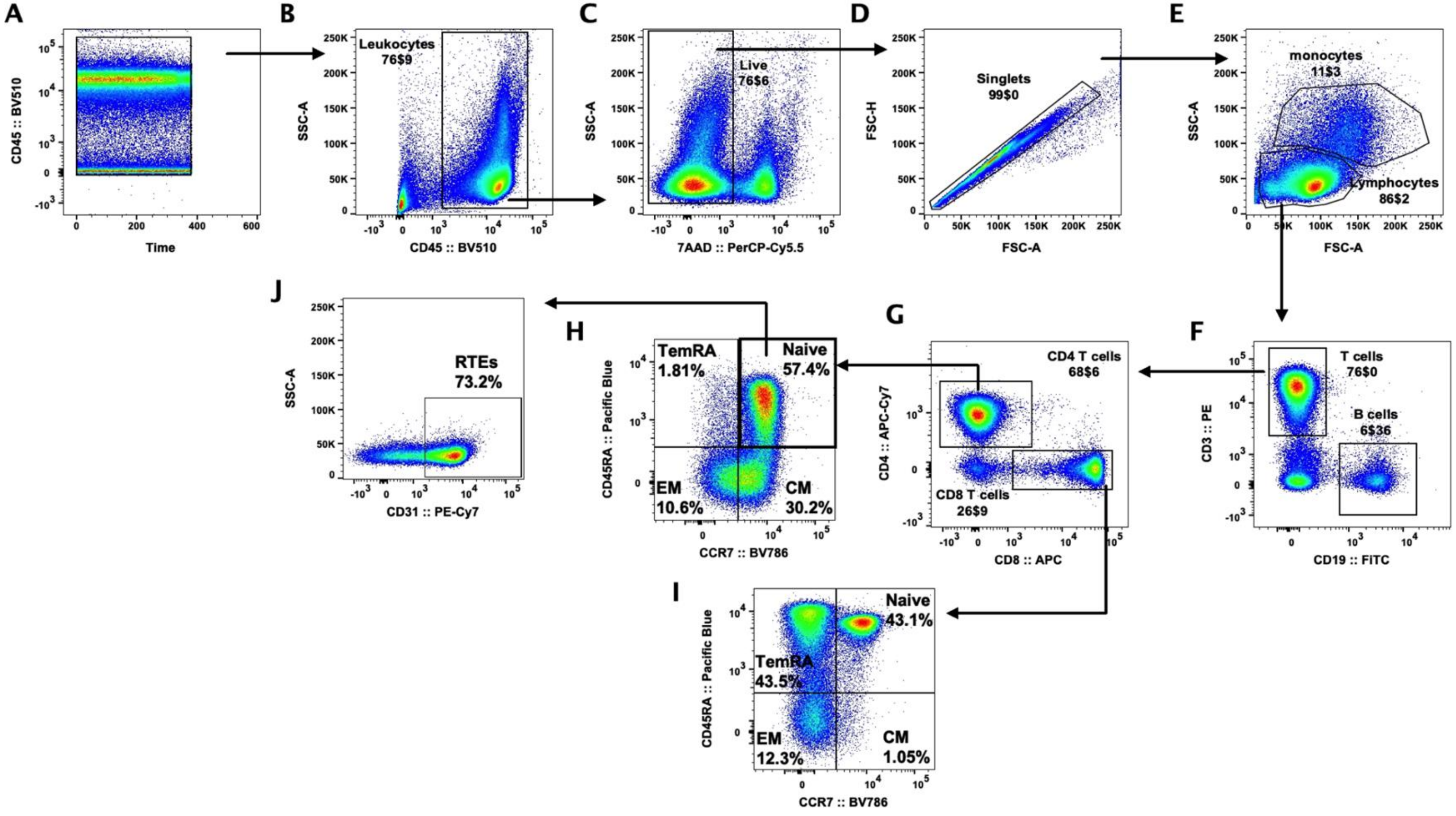

Supplementary Figure S4. Gating strategy for phenotypical analysis of the main T cell subsets. Issues during acquisition were verified by accompanying acquisition throughout time **(A).** Leukocytes were selected by their positive expression of CD45 (**B**), and viable cells selected by excluding 7AAD positive cells (**C**). After isolating the singlets (**D**), the monocytes and lymphocytes were gated based on their size (FSC-A) and complexity (SSC-A; **E**). Within the lymphocytes population, B cells were distinguished from T cells, through the differential expression of CD19 and CD3, respectively (**F**). $CD4^+$ and $CD8^+$ cell subsets were then discriminated among T cells (**G**). CD45RA and CCR7 were used both for $CD4^+$ (**H**) and $CD8^+$ (**I**) T cells to discriminate the Naïve ($CD45RA^+CCR7^+$), Central Memory (CM; $CD45RA^-CCR7^+$), Effector Memory (EM; $CD45RA^-CCR7^-$) and CD45RA-expressing memory cells (TemRA; $CD45RA^+CCR7^-$) subsets [**H** and **I**]. Total memory $CD4^+$ or $CD8^+$ T cells were the sum of CM, EM and TemRA cells. Finally, recent thymic emigrants (RTEs) were defined by the expression of the CD31 among naïve $CD4^+$ T cells ($CD45RA^+CCR7^+CD31^+$; **J**).

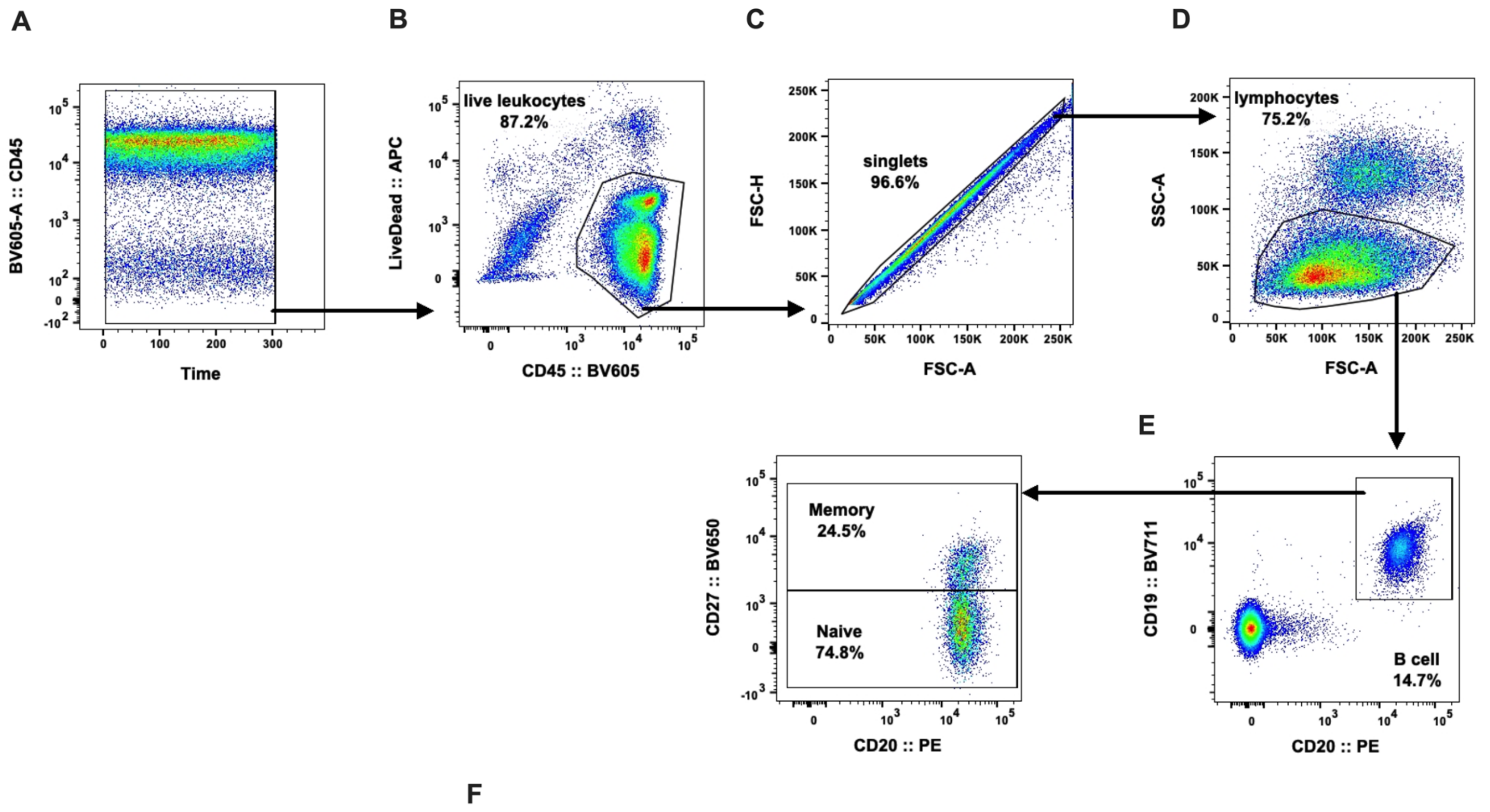

Supplementary Figure S5. Gating strategy for analysis of B cell subsets. Any acquisition issues were controlled by checking acquisition throughout time (**A**). Live leukocytes were defined based on negative staining for the viability dye and CD45 positive expression (**B**). Following singlet isolation (**C**), size (FSC-A) and complexity (SSC-A) gating was used to define lymphocytes (**D**). Within the lymphocyte population, B cells were defined as $CD19^+CD20^+$ (**E**). According to CD27 expression the naïve ($CD27^-$) and memory ($CD27^+$) $CD20^+$ B cell subsets were defined.

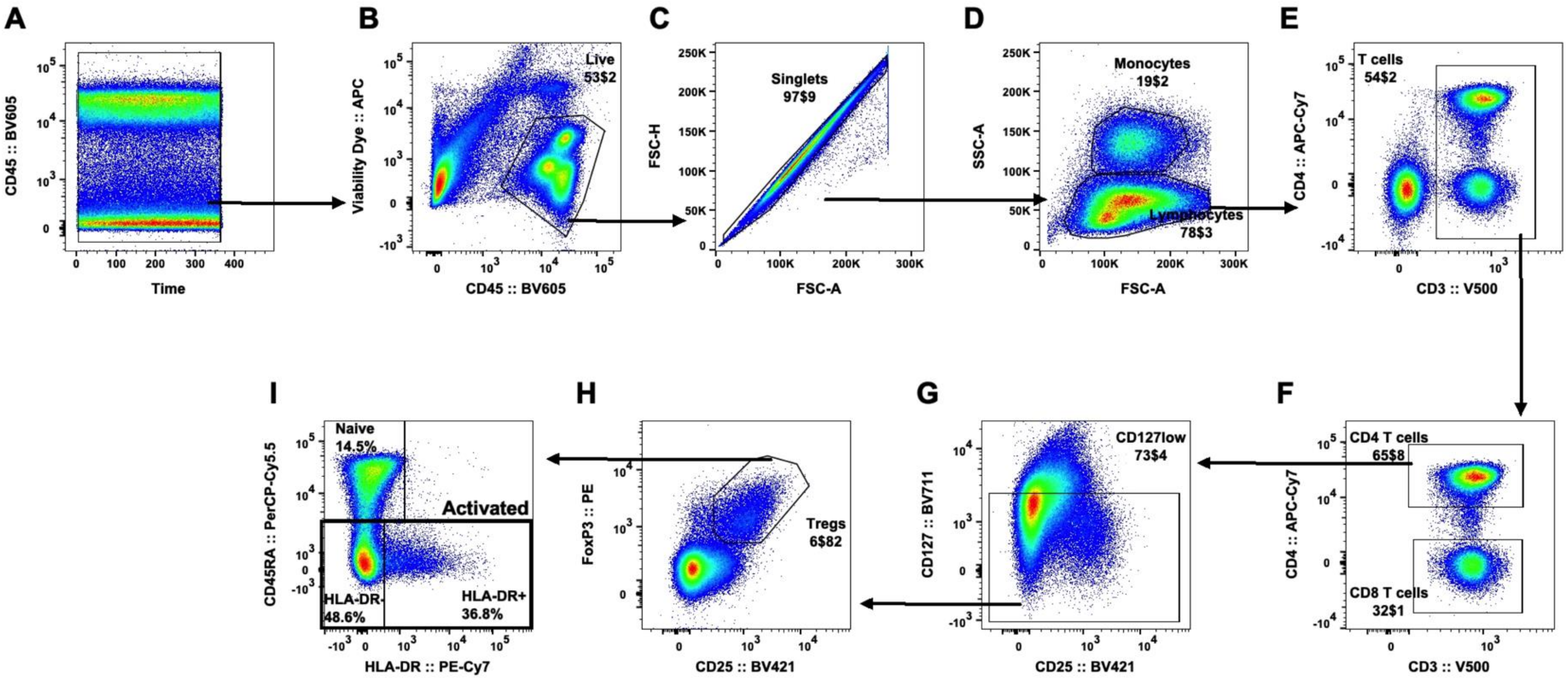

Supplementary Figure S6. Gating strategy for phenotypical analysis of the regulatory T cells (Tregs). Issues during acquisition were verified by accompanying acquisition throughout time **(A).** Live leukocytes were selected by their positive expression of CD45 and by excluding positive cells for the viability dye (**B**). After isolating the singlets (**C**), the monocytes and lymphocytes were gated based on their size (FSC-A) and complexity (SSC-A; **D**). Within the lymphocytes the T cells were defined according to the positive expression of CD3 (**E**). The population $CD4^+$ T cells was then defined by its positive expression of CD4 (**F**). Tregs were defined as the $CD4^+$ T cells expressing low levels of CD127 (**G**), followed by selection of $FoxP3^+$ $CD25^+$ cells (**H**). Finally, the CD45RA and HLA-DR markers were used to define three Tregs subsets: the naive ($CD45RA^+HLA\text{-}DR^-$), the activated $HLA\text{-}DR^-$ ($CD45RA^-HLA\text{-}DR^-$) and the activated $HLA\text{-}DR^+$ ($CD45RA^-HLA\text{-}DR^+$) Tregs (**I**). Total activated Tregs was the sum of activated $HLA\text{-}DR^-$ and $HLA\text{-}DR^+$ cells.

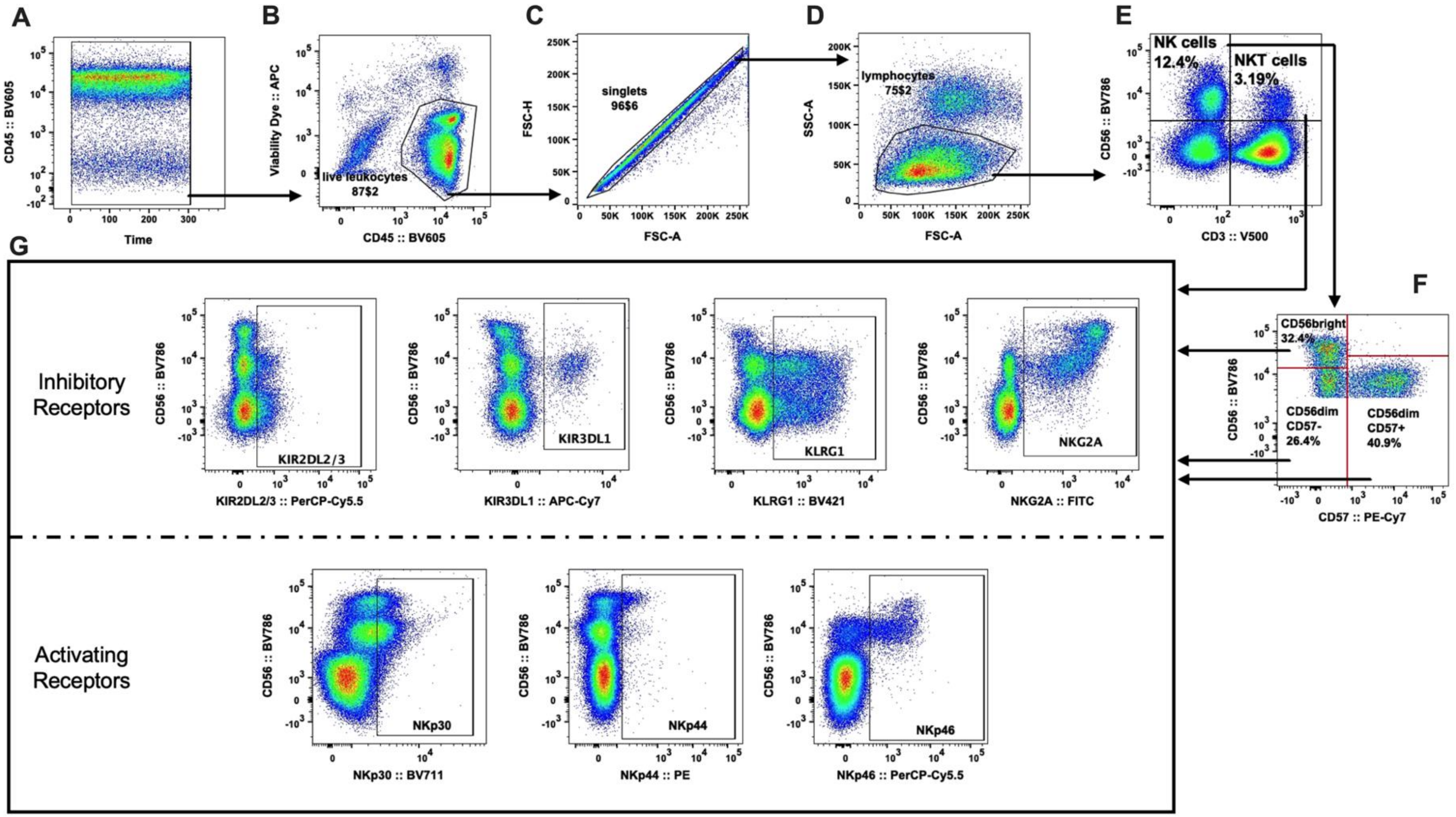

Supplementary Figure S7. Gating strategy for analysis of inhibitory and activating receptor expression on NKT and NK subsets. Any acquisition issues were controlled by checking acquisition throughout time (**A**). Negative staining for the viability dye and CD45 positive expression were used to define live leukocytes (**B**). Following singlet isolation (**C**), size (FSC-A) and complexity (SSC-A) gating was used to define lymphocytes (**D**). Within the lymphocyte population, NK and NKT cells were defined as $CD56^{+}CD3^{-}$ and $CD56^{+}CD3^{+}$, respectively (**E**). Three main NK cell subsets were discriminated according to CD56 and CD57 expression levels: $CD56^{bright}$ NK cells, $CD56^{dim}CD57^{-}$ NK cells and $CD56^{dim}CD57^{+}$ NK cells (**F**). Inhibitory (KIR2DL2/3, KIR3DL1, KLRG1 and NKG2A) or activating receptor (NKp30, NKp44 and NKp46) expression were assessed within all NK subsets and NKT cells, according to the staining panel (**G**).
